# The Effectiveness of Video Visual Scene Display-Assisted Behavioral Skills Training in the Instruction of Skills to Prevent Online Sexual Abuse Among Adults with Autism Spectrum Disorder

**DOI:** 10.1007/s10508-025-03152-z

**Published:** 2025-05-30

**Authors:** Çimen Oğur, Seray Olçay

**Affiliations:** 1 Department of Special Education, Faculty of Education, Hacettepe University, 06800 Beytepe/Ankara, Turkey; 2 Department of Special Education, Ankara University Faculty of Educational Sciences, 06590 Cebeci, Ankara, Turkey

**Keywords:** Autism spectrum disorder, Online sexual abuse, Visual scene display, Behavioral skills training

## Abstract

The present study aimed to determine the effectiveness of the video visual scene display-assisted behavioral skills training in skills to prevent online sexual abuse among adults with autism spectrum disorder (ASD). The study was conducted with three 21–23-year-old male individuals with ASD, and the multiple probe model across participants, a single-subject research model. The study findings demonstrated that all individuals acquired the skills to prevent online sexual abuse, generalized the acquired skills to different individuals, and maintained these skills for 2–4 weeks after the instruction. Furthermore, social validity data were collected from the participants, their parents, and teachers with the subjective analysis approach, and it was observed that participants, their parents, and teachers reported positive views about the instruction of the skills, the intervention, and the outcomes.

## Introductıon

Individuals with ASD are four to eight times more exposed to sexual abuse when compared to individuals with typical development (McDaniels & Fleming, 2016) due to communication difficulties such as denial or asking for help (Edelson, 2010), inability to recognize the social cues that could allow them to differentiate safe and unsafe behavior (Ballan & Freyer, 2017), problems associated with social-cognitive skills such as social problem solving and decision-making (Good & Fang, 2015), and cognitive theory skills that could allow comprehension of different perspectives (Carlson et al., 2013).

On the other hand, it was emphasized in the literature that internet use (Macmillan et al., 2022; Normand & Sallafranque-St-Louis, 2016), access to social media and game sites to socialize and share their interests using technological devices such as smartphones, tablets or computers of individuals with ASD, has increased (Mazurek et al., 2015; van Schalkwyk et al., 2016), which in turn increased their exposure to online sexual messages and posts (Normand & Sallafranque-St-Louis, 2016) and, therefore, to online sexual abuse. Thus, the instruction of skills to prevent online sexual abuse is essential to individuals with ASD (Nichols & Blakeley-Smith, 2009). However, the literature review revealed no research on the instruction of skills to prevent online sexual abuse to individuals with ASD (Miltenberger & Novotny, 2022; Tekin-İftar et al., 2021), leading to a critical need to determine effective interventions for the instruction of these skills.

Behavioral skills training (BST) is an effective method to instruct safety skills to individuals with ASD (Miltenberger, 2008; Miltenberger et al., 2015). In a meta-analysis conducted on the single-subject research that focused on the instruction of safety skills to individuals with ASD, Tekin-Iftar et al. (2021) reported that BST was an evidence-based practice for the instruction of various safety skills to individuals with ASD. BST is an instruction method that includes four stages: identification of security threats and instruction of adequate behavior against these threats (instruction), demonstration of adequate behavior (modeling), performing roles that include adequate behavior (rehearsal), and feedback (Miltenberger, 2008). The review of the studies on BST in the instruction of safety skills revealed that BST has been employed to instruct skills to protect 4–13-year-old individuals with ASD from abduction-prevention skills (Gunby & Rapp, 2014; Johnson et al., 2005), to display safe behavior during a fire (Garcia et al., 2016) and against firearms (Gross et al., 2007), to cross the street (Goldsmith, 2008), to open the door when the doorbell rings (Summers et al., 2011; Tavukçu, 2021), and to protect personal information online (Zinicola, 2021). However, there are studies in the literature that investigated the effectiveness of BST in the instruction of skills to prevent sexual abuse to participants with intellectual disabilities (Egemo-Helm et al., 2007; Lee & Tang, 1998; Lumley et al., 1998; Miltenberger et al., 1999). Egemo-Helm et al. (2007) studied 28–47-year-old women with intellectual disabilities, Lumley et al. (1998) studied 30–42-year-old women, and Miltenberger et al. (1999) studied 33–57-year-old women with intellectual disabilities. The findings of these studies conducted with the multiple baseline model, a single-subject research design, demonstrated that BST was effective in the instruction of skills to prevent sexual abuse to certain adult women with intellectual disabilities, while further training was required for others. Although these studies also collected follow-up and generalization data, participant performances differed. Furthermore, the social validity data collected in two studies (Egemo-Helm et al., 2007; Lumley et al., 1998) demonstrated that the views of the participants who included program managers and colleagues were positive about the instructed skills, BST, and the outcomes. In contrast, Lee and Tang (1998) studied 72 11–15-year-old adolescents diagnosed with mild intellectual disabilities. The study data were collected with pre-test, post-test, and follow-up sessions conducted with experimental and control groups. BST was employed to instruct the participants the skills to prevent sexual abuse in the experimental group, embracing instruction, modeling, rehearsal, social reinforcement, and feedback were employed in the control group to instruct safety skills such as car, classroom, and water safety skills, which were not associated with the skills to prevent sexual abuse. The study findings demonstrated that the knowledge of the participants in the experimental group about sexual abuse and self-protection improved in the post-test, and retained for two months.

Although the efficacy of BST in the instruction of skills to prevent sexual abuse to individuals with intellectual disabilities has been demonstrated in the literature, there is a need for further studies to demonstrate its efficacy in the instruction of skills to prevent sexual abuse to individuals with ASD. Furthermore, we could not identify a study on the effectiveness of BST in the instruction of skills to prevent online sexual abuse. Furthermore, about 45% of the studies on the effectiveness of BST in the instruction of safety skills to individuals with ASD collected follow-up, generalization (Tavukçu, 2021), and social validity (e.g., Johnson et al., 2005) data, while the need for research where follow-up, generalization, and social validity data are collected continues.

The review of the studies on the effectiveness of BST based on method steps revealed that both video and live models were employed in the modeling stage (Ledbetter-Cho et al., 2021), and these studies reported that video models led to the most accurate display of the skills and were recommended for the instruction of safety skills (Miltenberger, 2008; Olcay et al., 2023). Previous studies also emphasized that video model-assisted instruction was preferred by individuals with ASD (Shane & Albert, 2008), and these approaches facilitated the acquisition of various skills (i.e., academic, communication, vocational, leisure, and safety skills) during instruction (Kagohara et al., 2013). The studies emphasized the significance of the support of the videos by hot spots, texts, and sounds that led to the most effective outcomes (Light et al., 2019; Miltenberger & Novotny, 2022).

Visual scene display (VSD) instruction entails the development of video for the targeted skill, where the video display is paused to demonstrate how the skill should be exhibited, the relevant image is circled, and the textual or audio outputs about the emphasized points become active on the display. The application improves the interaction of the individual with the video (Light et al., 2014). Due to these features, research demonstrated that video VSD application helps 3–21-year-old individuals with ASD to acquire social interaction (Chapin et al., 2022), social adaptation (Laubscher et al., 2019), and vocational skills (Babb et al., 2021), and it was effective in conceptual instruction (Holyfield et al., 2020); however, it also evidenced the need for further studies on the effectiveness of video VSD in the instruction of various skills to individuals with ASD.

Consequently, it was determined that (1) individuals with ASD, who experience difficulties in recognizing security threats, displaying safe behavior in these situations, and reporting these situations, are among the high-risk individuals for sexual abuse, (2) the popularity of the internet use increased the presence of individuals with ASD on the internet and their vulnerability to online sexual abuse, (3) there are no studies on the instruction of skills to prevent online sexual abuse to individuals with ASD, (4) there is a need for the determination of effective interventions for the instruction of skills to prevent online sexual abuse to individuals with ASD, and (5) there was no study where the effectiveness of an intervention where BST and the video VSD were employed in the modeling step in the instruction of skills to prevent online sexual abuse. The present study, based on the points emphasized in the literature, aimed to investigate the effectiveness of video VSD-assisted BST in the instruction of skills to prevent online sexual abuse among adults with ASD. Thus, the following research questions were determined.

1. Is video VSD-assisted BST effective in the acquisition of skills to prevent online sexual abuse by individuals with ASD?
2. Can individuals with ASD maintain the skills to prevent online sexual abuse for 2–4 weeks after the training if they acquire these skills?
3. Can individuals with ASD generalize the skills to prevent online sexual abuse to other individuals if they acquire these skills?
4. What are the views of individuals with ASD, their parents, and teachers about the target skill, the method employed for the instruction of this skill, and the study findings?

## Method

### Participants

The study was conducted with three 21–23-year-old individuals with ASD. All participants were male. Participants over the age of 18 who attended wellness centers, foundations, special education, and rehabilitation centers were determined. The participants were assigned based on their medical board reports. The parents of the 18 preselected individuals were interviewed individually to inform them about the study. After these interviews, 12 parents who volunteered the participation of their children were contacted and preliminary data were collected about their children. Preliminary data included smartphone and social media use of the children. Thus, seven participants who did not use smartphones and social media were excluded from the study. The parents of the remaining five participants signed the informed consent form, and these participants were evaluated to determine whether these individuals met the prerequisite skills for the study. The three participants and their families who met the prerequisites were informed verbally and in writing about the aims, importance, and processes of the study. They were asked to sign a consent form indicating that they wanted to voluntarily participate in the study. In addition, code names were determined for the participants to protect personal information. Detailed information about these prerequisite skills and how these skills were evaluated are presented in the following paragraph.

(1) The participants were instructed to complete simple tasks such as taking their book from the bookshelf and putting it on the table to evaluate their ability to follow two-step instructions. Four trials were implemented, and it was decided that the participants exhibited this skill when they completed each instruction within five seconds. (2) To assess their ability to imitate at least two-step tasks and verbal expressions, the author sat with the participants and asked them to do what she did. After the instruction, their behaviors (i.e., drinking coffee, putting their hand on their head) and whether they imitated the expressions (i.e., phone key lock, theater stage) were observed. Two four-trial sessions were conducted. In one of the sessions, the participants were expected to imitate the exhibited behavior and expressions within five seconds, and when the participants imitated all behaviors and expressions, it was accepted that they exhibited the skill. (3) Videos were displayed to determine the ability of the participants to concentrate on visual and auditory stimuli for at least five minutes. Whether they concentrated on the video was determined with momentary time sampling. Five minutes of observation was divided into 1-min sections. When the participants could concentrate on the video for the whole 1-min interval, it was decided that the participants exhibited the skill. (4) To assess their ability to distinguish the private part of the body, participants were asked to tell “our private areas,” and the participants were expected to respond correctly within five seconds. A four-trial session was conducted to evaluate the skill. When the participants could tell all private areas correctly within five seconds in all four trials, they were accepted to exhibit the skill. (5) To determine whether the participants could identify sexual messages, they were presented with five non-sexual messages (e.g., “I am very curious about the book you are reading. Can you send me a picture of the book you read?”) and five sexual messages (e.g., “You’ve got a nice body. Take off your clothes and send me a picture. I want to see you.”), and it was determined that the participants possessed these skills when they could identify all massages accurately. (6) To determine the self-expression skills with at least two words, the participants were asked daily life questions such as “What did you do today?” in a four-trial session. The participants were asked four questions, and they were considered to possess this skill when they responded to all questions with at least two-word sentences. (7) To evaluate their reading, comprehension, and writing skills in at least two-word sentences, the participants were asked to read sentences such as “The weather is very nice today,” and then respond to two questions about the sentence in a four-trial session. Then, a second four-trial session was conducted, and the participants were asked to write down the sentences that were read by the author. When they could read the sentences in all trials, answer all questions, and write all sentences, they were considered to possess these skills. (8) To evaluate the skill to effectively use at least one social media account, the participants were asked to send a message, make a video call, or block an account in their social media accounts (e.g., WhatsApp, Instagram) in a session where one trial was devoted to each skill. The individuals with ASD were considered to possess these skills when they performed 100% of all three skills.

Bruno was a 21-year-old male diagnosed with ASD. He was attending a special education and rehabilitation center (an institution specialized in the education of individuals with ASD) that served different age and diagnosis groups. Bruno could read and write independently, recognize private areas, and use social media tools such as WhatsApp, but could not respond correctly to inappropriate messages online.

Dan was a 22-year-old male diagnosed with ASD. He was attending an educational foundation that served individuals with ASD in different age groups. He could read and write independently, and use social media tools such as WhatsApp, but could not respond correctly to inappropriate messages online.

Adam was a 23-year-old male diagnosed with ASD. He was attending a municipal institution for the education of individuals of different ages and diagnostic groups. Adam could read and write independently, establish causality, and use social media tools such as WhatsApp, but could not respond correctly to inappropriate messages online. Participant names are pseudonyms.

#### Practitioner

All sessions were conducted by the first author. The practitioner is a graduate student who graduated from a special education undergraduate program and has 9 years of experience with individuals with special needs. She also completed an Autism and Behavioral Sciences program in Canada.

#### Observer

Reliability data for the dependent and independent study variables were collected by a Ph.D. candidate in the Department of Special Education. Before the reliability data were collected, the observer was informed by the practitioner about the aim of the research, dependent and independent variables, and data collection forms.

#### Peer Models

Peer models played in the videos developed for the second step of BST, the modeling stage. In this stage, four individuals with typical development, 18–36-year-old two females and two males performed as peer models. Peer models were previously unknown by the participants. For them to exhibit the target skill accurately and completely, they were trained in BST stages that included the instruction, modeling, rehearsal, and feedback components. In the training, they were instructed about the type of reactions they should exhibit when they received a sexual message on WhatsApp from a sexual perpetrator. The practitioner demonstrated how the models should behave when they receive a sexually explicit message on WhatsApp, namely the “no/not respond (reject the message by typing no or ignore the message),” “block (block the number),” and “report (to a trusted adult)” steps. In the rehearsal step, the peer models were expected to exhibit the above-mentioned three steps after they received a message on WhatsApp. In the feedback stage, the peer model responses in the rehearsal stage were observed and feedback was provided by the practitioner about their performance. This practice continued until the models were 100% accurate in the rehearsal stage.

#### Perpetrators

Thirty-nine individuals, 18–54-year-old 11 men and 28 women, participated as online perpetrators in the baseline and intervention sessions. Online perpetrators for the baseline, intervention, and follow-up sessions were selected from individuals of different ages, genders, and physical attributes, who were unknown by the participants. For the generalization sessions, perpetrators were selected from individuals whom the participants had met before but did not encounter in daily life (i.e., sister’s girlfriend, father’s colleague).

Written informed consent was obtained from peer models and perpetrators to document voluntary participation, and they signed a contract that they would only communicate with the participants during the study at the time and for the content specified by the practitioner, and they would keep participant information confidential.

### Setting

The baseline, baseline, follow-up, and generalization sessions were conducted online, and the practice sessions were conducted in the educational institutions that the participants attended. Interviews were conducted with the parents to determine the online environment. One parent stated that their child used WhatsApp and Instagram, and two parents stated that their child used WhatsApp. Thus, WhatsApp was determined as the study application, which was used by all participants.

### Materials

**Fig. 1:**
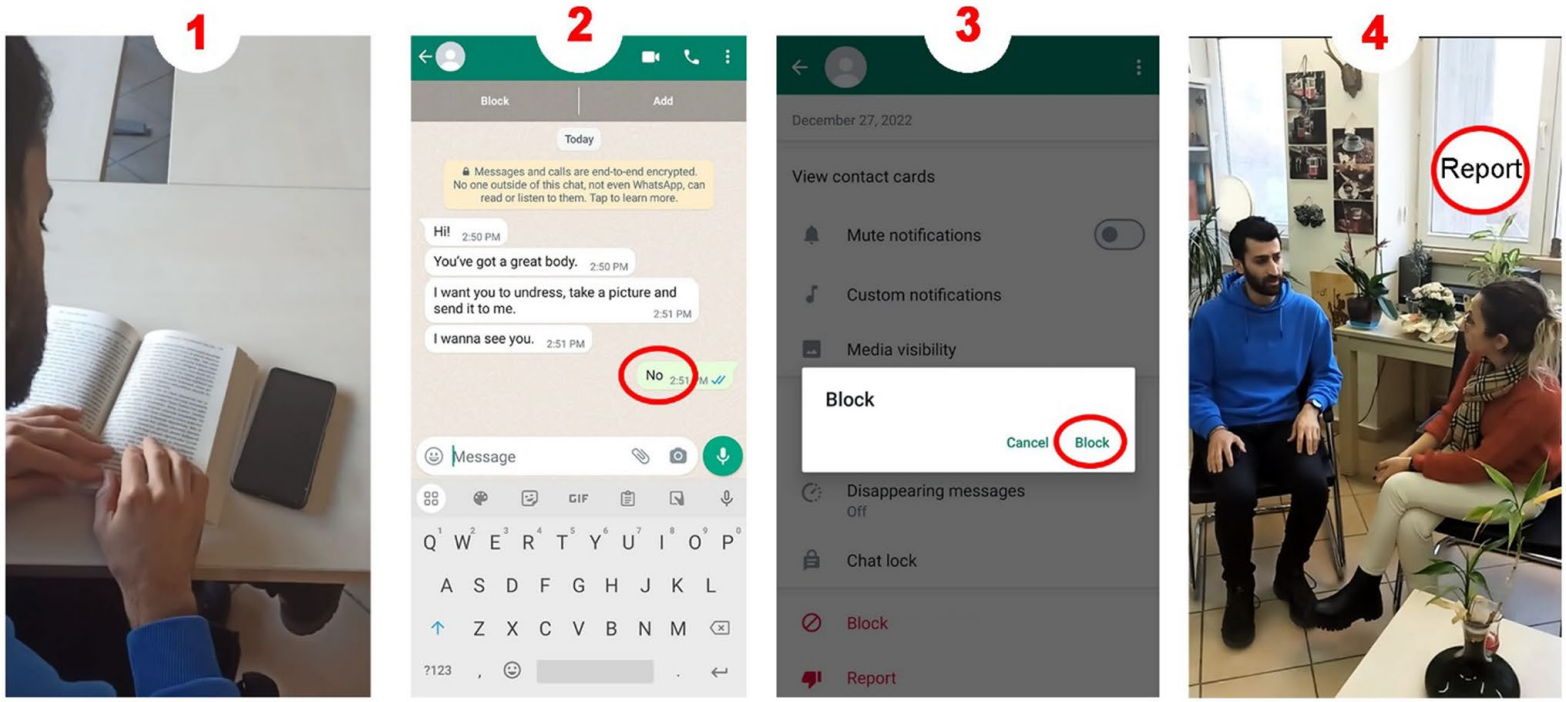
Screenshots of the visual displays

In the study, the effectiveness of the video VSD-assisted BST in the instruction of skills to prevent online sexual abuse was investigated. Thus, four videos that lasted an average of 2 min and 30 s (between 1 min 39 s and 3 min 12 s) based on VSD features and various scenarios were developed. The videos featured peer models and perpetrators. Furthermore, individuals who portrayed trusted individuals, such as parents, elder sisters, and teachers, also participated in the videos. One of the videos was as follows: As the participant read a book, a message arrived on WhatsApp from an unknown individual. The message from the perpetrator read as follows: “You’ve got a nice body. Take off your clothes and send me a picture. I want to see you.” Then, the participant typed “No” to the system message, blocked the number of the perpetrator, and reported the event to the adult who played his mother. Two videos included the “rejection,” “blocking,” and “reporting” steps, while two videos included “not responding/ignoring,” “blocking,” and “reporting” steps. After the videos were shot based on the scripts, VSD features were added to each video using the following three steps: addition of the hot spots, recording the hot spots, and activation of the hot spots. In these videos, pauses were included in the scenes that aimed at the instruction of the skills. The video is paused to display a still photo that demonstrates the skill. This image is circled and the hot spots that would allow a printout were recorded on the image. Samples are presented in Fig. 1.

Furthermore, a laptop computer was used to allow the perpetrators to watch the VSD videos that included 19 scripts, and data collection forms were employed to record the performances of the participants and the practitioner. The scripts and data collection forms were reviewed by three experts, one of whom was a teacher and two of whom were faculty members at the special education department. The scripts and forms were finalized based on the feedback of the experts.

### Measures

The dependent variable of the study was the level of acquisition of skills to prevent online sexual abuse by individuals with ASD. WhatsApp, the common application platform in the study, was the online medium. Two response types were defined in the study: correct and incorrect responses. The correct completion of the rejection or ignorance, blocking the perpetrator, and reporting the event stages within five minutes upon receipt of the online sexual message was considered the correct response, and a (+) sign was noted in the data collection form for that trial. The inability of a participant to exhibit any of these steps or incorrect participant action in any of the steps was considered as an incorrect response, and a (−) sign was put in the data collection form for that trial. The independent variable of the study was the video VSD-assisted BST. The BST steps, namely the instruction, modeling, rehearsal, and feedback steps, were conducted in the study (Miltenberger, 2008). The modeling step was conducted with the video VSD application.

### Procedure

In the study, baseline, intervention, follow-up, and generalization sessions were conducted. Two sessions were conducted three times a week, and different scripts were employed in each session. The scripts were developed based on the opinions of the parents, and the messages that the participants received in daily life or were likely to receive. Furthermore, the scripts were revised based on parental requests about novel messages or messages that would likely trap the participants. Expert opinion was consulted after the revisions.

#### Baseline

Baseline sessions were conducted simultaneously for all participants within five sessions. Four trials were conducted in each baseline session. Furthermore, all baseline data were collected with online in situ assessment. Thus, data collection was conducted in coordination with the parents when it was convenient for the parents. Each trial started by sending a sexual abuse message to the participant on WhatsApp. The participants were expected to type “No” or ignore the message, block the online perpetrator, and report this issue to a trusted adult within five minutes when they saw the message. The rejection or ignorance of the message was determined by examining the WhatsApp correspondence of the perpetrator. These individuals were asked to provide a screenshot of the correspondence to the practitioner. To determine whether the participants blocked the number of the perpetrator, a second message was sent to the individuals to determine whether the second message could be delivered. It was accepted that the number of the perpetrator was blocked when the message could not be delivered (a single checkmark next to the message sent). The step of reporting the event to a trusted adult was determined based on parental feedback. The same procedure was conducted in all four trials. The correct response rate was calculated with the formula [(Number of trials with correct response/Total number of trials) X 100]. The results were plotted on a graph. In the study, trusted adults included the parents, the author, and the institution principal. Data collection continued until stable data were collected from the participants in at least three consecutive sessions.

#### Intervention Sessions

The intervention sessions were conducted with video VSD-assisted BST. Sessions were planned as three days and two sessions per week. One of the videos was selected randomly for each session; however, the inclusion of all videos was ensured. The intervention sessions started with the instruction step in BST. In this step, the participant was instructed that when he recognizes an online sexual message, it is very important that he should reject the message by typing “No” or ignore the message, and report it to a trusted adult for safety, and all these steps are demonstrated. Then, in the modeling step, the practitioner stated that s/he developed a video about dealing with online inappropriate messages and they could watch the video together. Thus, the practitioner raised the interest of the participant about the target skill and the video. Then, the participant was instructed to watch carefully and allowed to watch one of the randomly selected videos. After reinforcing the participant to carefully watch the video, the rehearsal step was initiated. In this step, a sexual message was sent to the participant on WhatsApp, providing a natural opportunity for the participant to exhibit the learned correct response. Correct participant responses were reinforced, and corrective feedback was provided in case of an incorrect response. For instance, when the participant rejected the message; however, failed to block the perpetrator and report the event to a trusted adult, the practitioner intervened as follows: “It was correct to reject this message, but you also needed to block the person who sent the message and report the message to a trusted adult. Let us repeat these steps.” The rehearsal and feedback steps were repeated until the response was 100% correct in the rehearsal step. Furthermore, the correct responses of the participants were reinforced at all steps.

In intervention sessions, the practitioner was expected to (1) instruct the target behavior (behaviors that should be exhibited to cope with online sexual abuse) and the significance of the target behavior, (2) provide the model by allowing the participant to watch the video that displays the target behavior, (3) conduct the rehearsal of the target behavior by creating natural opportunities, (4) provide feedback about participant’s performance during the rehearsal (reinforce correct responses, provide corrective feedback for incorrect responses) (Miltenberger, 2008).

#### Follow-Up Sessions

Follow-up sessions were conducted 2 and 4 weeks after the intervention sessions. Follow-up sessions were similar to the baseline sessions.

#### Generalization Sessions

In the baseline and intervention sessions, the messages based on various scripts were sent by various unknown perpetrators. Thus, measures were adopted to ensure generalization during the intervention. Generalization data across people were also collected in the study with generalization pre-test and post-test sessions. In these sessions, online sexual messages were sent by familiar perpetrators. To allow the participants to continue healthy relations with people they frequently encounter in daily life, familiar perpetrators were individuals whom the participants had met before but did not encounter with these individuals in daily life. For this purpose, interviews were conducted with the parents of the participants and individuals whom the participants met before but were not likely to meet again. For example, in the case of Bruno, an interview was conducted with his sister's college friend, who lived in another city, and with whom even his sister could only communicate online. In the case of Dan, an interview was conducted with a friend of his mother, who was a previous neighbor but moved to a different country about 3 years ago. And finally, in the case of Adam, an interview was conducted with a previous colleague of his father, who now lives in another city. Furthermore, these individuals were initially contacted by the parents, and the interviews were conducted with parental approval due to ethical concerns.

### Data Analysis

In the study, reliability, effectiveness, and social validity data were collected.

#### Reliability

Inter-observer reliability and treatment reliability data were collected in 50% of the baseline, intervention, follow-up, and generalization sessions. Initially, the order numbers were assigned to sessions reliability calculations were conducted on the neutral session videos and WhatsApp correspondence screenshots. No session data were provided to the observer for the videos and correspondence screenshots. The formula [(the number of agreements / the sum of both agreements and disagreements) X 100] was used to analyze the inter-observer reliability data (Watkins & Pacheco, 2000). The inter-observer reliability rate was calculated as 100% for all individuals with ASD. The treatment reliability data were analyzed with the [(the number of times the implementer correctly performed the step/ the number of opportunities to perform the step) X100] formula (Ledford & Gast, 2014), and it was determined that the practitioner exhibited 100% accuracy in all performances conducted with all participants.

#### Effectiveness

In the study, intervention efficacy data were collected based on participant performances in baseline, intervention, follow-up, and generalization sessions. The data were analyzed with graphs. The efficacy data were also analyzed visually. In the visual data analysis, six properties were analyzed: level, trend, stability, immediacy of effect, non-overlap, and consistency of data patterns across similar phases (Kratochwill et al., 2013). Furthermore, the effect size was calculated with the improvement rate difference (IRD). In the study, the progression bar was employed to determine the data trend (Lane & Gast, 2014). The formula suggested by Lane and Gast (2014) was employed to calculate stability. Based on the formula, + 25% of the median of the intervention phase was taken. Then, the number of data points left was divided by the total number of data points, and the result was multiplied by 100. The formula proposed by Aydin and Tanious (2022) was employed to determine the immediacy of the effect. Based on the formula, the arithmetic mean of the first three data points in the intervention phase was taken. This value was divided by 100, which gave the success criterion determined for the participants. To calculate the percentage of non-overlapping data, the number of data points outside the range of the baseline phase was determined in the intervention phase and divided by the total number of data points in the intervention phase, and the result was multiplied by 100 (Tekin-Iftar et al., 2019). IRD was determined with the online tool (www.singlecaseresearch.org/calculators/ird) developed by Vannest et al. (2011).

#### Social Validity

In the study, social validity data were collected with the interviews conducted with the participants, their parents, and teachers and the subjective analysis approach. Thus, a social validity survey was developed and employed by the authors. The participant survey included five closed-ended and four open-ended and a total of nine questions. The parental and teacher survey included nine open-ended questions. The social validity data were analyzed descriptively. The survey forms are presented in Table 1.

**Table 1:** Social validity forms

Social validity form for individuals with ASD		Social validity form for parents and teachers
1. We learned how to protect ourselves against inappropriate messages on WhatsApp. Did you enjoy learning this skill?	□ Yes □ No	1. Do you think it is important to instruct the skills to prevent online sexual abuse to individuals with ASD? Please elaborate
2. Do you think you feel safer after learning these skills?	□ Yes □ No	2. Do you think the instruction of skills to prevent online sexual abuse to individuals with ASD would contribute to independent living? Please elaborate
3. Do you think learning these skills made people around you happy?	□ Yes □ No	3.What do you think about the method used for the acquisition of these skills?
4. Did you communicate comfortably with your teacher when learning this skill?	□ Yes □ No	4. What do you think about the videos employed during training?
5. Did you like the way your teacher instructed this skill?	□ Yes □ No	5. Do you think that individuals with ASD will use the skills they learned after the training?
6. Would you like to learn another skill this way? Why?	□ Yes □ No	6. It was observed that individuals with ASD generalized these skills to other individuals after the training. What do you think about this? Which properties of the method do you think contributed to this effect?
7. Do you think learning this skill will make your life easier? Why?		7. What did you like about the study?
8. What did you like about the study?		8. What did you dislike about the study?
9. What did you dislike about the study?		9. Did you think before the study that the student/child could acquire these skills?

## Results

**Fig. 2:**
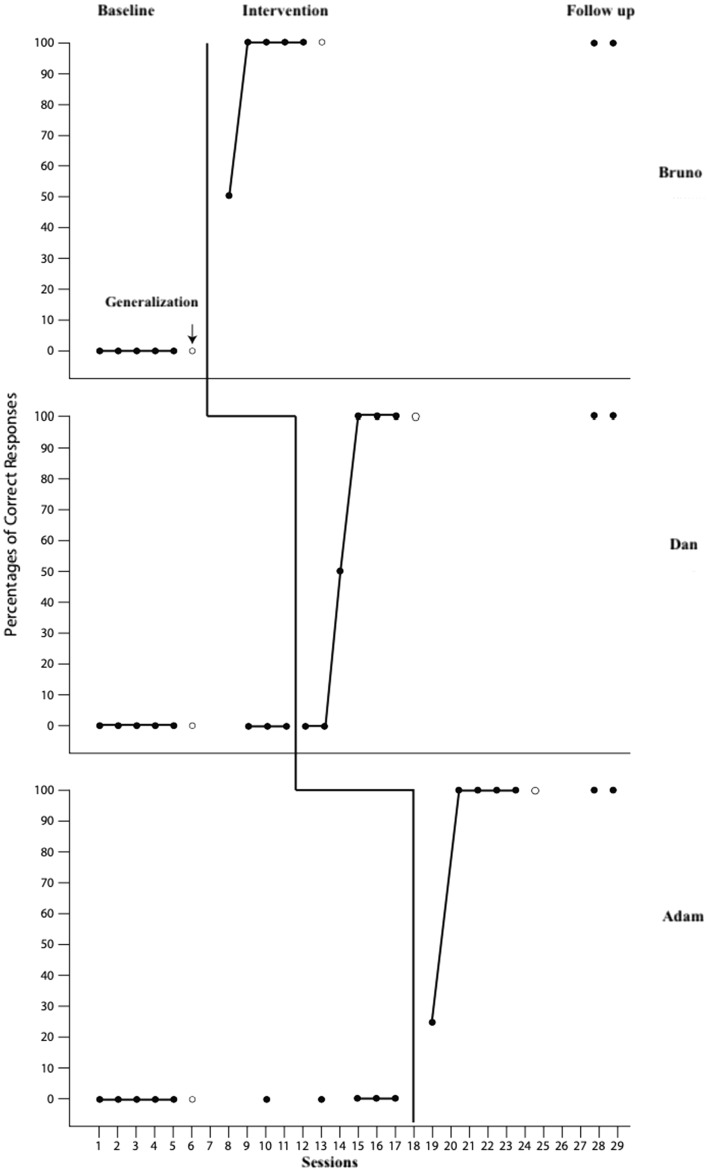
Performance of the participants during the baseline, ıntervention, follow-up, and generalization

The baseline, intervention, follow-up, and generalization session performances were analyzed to determine the effectiveness of the video VSD-assisted BST. The findings are presented in Fig. 2. Visual analysis data are presented in Table 2.

**Table 2:** Visual analysis

Participants	Level	Trend	Stability	Immediate effect	Non-overlap	Data patterns
Bruno	Yes	Positive	High (80%)	No delay (83.33%)	Very effective (100%)	Yes
Dan	Yes	Positive	Moderate (66.67%)	No delay (16.67%)	Questionable (66.67%)	
Adam	Yes	Positive	High (80%)	No delay (75%)	Very effective (100%)	

As seen in Fig. 2, the prevention of online sexual abuse performances of all participants was 0% in the baseline; however, they achieved 100% performance in the intervention phase. Generalization data revealed that the prevention of online sexual abuse performances of all participants was 0% in the pre-test session, while it was 100% in the post-test session. Furthermore, we observed that the skills in the prevention of online sexual abuse was 100% for all participants in all four trials in the follow-up sessions conducted 2 and 4 weeks after the training.

As seen in Table 2, the desired change was observed in the level and trend for all three individuals diagnosed with ASD. This change demonstrated that the intervention was effective and produced the desired outcomes. It was observed that the data stability varied between 66.67 and 80% in the intervention phase. The data exhibited a high level of stability for Bruno and Adam and moderate level for Dan (Tekin-Iftar et al., 2023). The immediate effect of the transition from the baseline to the intervention phase was 83.33% for Bruno, 75% for Adam, and 16.67% for Dan (Aydın & Tanious, 2022). This finding revealed that a rapid change was observed in the dependent variable with the implementation of the independent variable for Adam and Bruno. The analysis of the percentage of non-overlapping data rate revealed that the percentage of non-overlapping data rate was 100% for Bruno and Adam, and 66.67% for Dan, demonstrating that the intervention was very effective for Bruno and Adam, but questionable for Dan (Scruggs & Mastropieri, 2001). Finally, in the study, IRD was employed to calculate the effect size of the video-assisted BST. The effect size of the intervention was calculated as 100% for Bruno and Adam and 66.67% for Dan. The intervention’s effect was large for Bruno and Adam and moderate for Dan (Parker et al., 2009).

### Social Validity

Social validity data were collected from the participants, their parents, and teachers in the study. All participants stated that they and all related individuals were satisfied with learning the skills instructed in the study, learning these skills made them feel safe, they could easily communicate with the practitioner during the instruction, they liked the way the skill was instructed, and they wanted to learn other skills with the same method. Participants also reported that they enjoyed certain aspects of the study. Bruno: “I liked the ‘no,’ ‘block,’ and ‘report’ rules.” Dan: “(I liked) watching videos on the computer.” Adam: “I learned to say no to the (caller) numbers I don't know. I feel more safe.” All participants stated that there was nothing they did not like about the study; however, Bruno did not like receiving messages from people he did not know: “… I didn't like these people.”

The social validity data were collected from all teachers, while only two parents provided social validity data (Bruno's mother and Adam's father). The parents and teachers were initially asked whether they considered the instruction of skills to prevent online sexual abuse to individuals with ASD important. Both teachers and parents considered the instruction important. Bruno's teacher: “I think that since they do not know the concept of sexual abuse, they can trust people easily. They do not realize that these people could harm them.” When asked whether the instruction of skills to prevent online sexual abuse to individuals with ASD would contribute to independent living, all teachers and Adam's father responded the question positively, while Bruno's mother argued that individuals with ASD were more likely to be exposed to sexual abuse due to the traits associated with their diagnosis, and studying with individuals with ASD would not be sufficient, and parents of these individuals and other members of the society should also be trained. Bruno’s teacher stated the following: “I think yes, though not for every child. Because it raises awareness of the individual.” When parents and teachers were asked about their views on the method employed for the acquisition of the skills, they generally stated positive views. Adam's father stated the following: “I find this absolutely right. Because my son is active on social media, I think this method would be beneficial.” Adam’s teacher: "I think it facilitated the instruction of the topic and improved comprehension. I can say that it included all the steps that I think could facilitate learning. Thanks to the method, the individual not only learns the required knowledge, but also how to perform a certain behavior, practice the behavior, and receive feedback. In other words, it kind of meets all learning stages.” When asked about their views on the educational videos developed for individuals with ASD, only the response of Bruno's teacher was negative. Bruno's teacher stated the following: “It could have been more descriptive for these individuals.” Other parents and teachers emphasized that the videos were clear and comprehensible. When asked whether the individuals with ASD would use these skills after the training, all participants said yes. Adam’s father: “Yes, I think so. As an experiment, I tested it and got a positive response from my child.” All parents and teachers responded positively to the question “It was observed that individuals with ASD generalized these skills to different cases and individuals after the training. What do you think about this? What features of the method do you think contributed to this?” Bruno's mother: “My son’s perspective on different messages that he received on WhatsApp changed. He began to read the messages more carefully. He responded (to those) after thinking about it.” The teachers emphasized the impact of the videos and the presentation of several examples. All parents and teachers responded positively to the question “Are there any aspects of the study that you liked? If so, what are they?” Adam's teacher included the visuals and the collaboration with the parents among the aspects they liked about the study. When asked about the aspects of the study that they did not like, both parents and teachers stated that there was nothing they did not like about the study. Adam's teacher: “I think that training should be longer and supported by occasional repetitions.” Finally, when asked whether they thought their child could learn the instructed skills at the beginning of the study, all parents and teachers stated that individuals with ASD could learn these skills. However, Bruno’s and Adam's teachers stated that they hesitated at the beginning. Adam’s teacher: “I thought it would be very difficult. I hesitated, frankly, I thought that it might not be possible. I thought the probability of failure was higher. I had my doubts about the instruction of the skills independently. I still remember the happiness I felt when I witnessed the acquisition of the skill by the student.” Bruno's teacher: "I thought (it was possible), but I was hesitant that he could achieve independence. Thank you for being successful in that as well.”

## Discussion

The effectiveness of video VSD-assisted BST in the instruction of skills to prevent online sexual abuse to 21–23-year-old individuals with ASD was investigated. Furthermore, social validity data were collected from the subjects, parents, and teachers of the subjects with the subjective analysis approach. The effectiveness findings of the study demonstrated that all participants acquired the skills to prevent online sexual abuse with video VSD-assisted BST, they could maintain these skills 2 and 4 weeks after the training without any additional session, and they could generalize these skills to different individuals. Although there was no study on the effectiveness of BST in the instruction of skills to prevent online sexual abuse to individuals with ASD, the present study findings were consistent with the findings of previous studies on the effectiveness of BST in the instruction of safety skills to individuals with ASD (Goldsmith, 2008; Johnson et al., 2005). Furthermore, unlike the previous studies on the effectiveness of BST, the participants did not require additional instruction in the follow-up and generalization sessions (Gunby & Rapp, 2014). This could be due to the fact that the study participants did not have a concomitant intellectual disability. Studies where the skills to prevent sexual abuse were instructed to individuals with intellectual disabilities reported that in situ assessment or additional training sessions were required to ensure follow-up and generalization (Egemo-Helm et al., 2007; Lumley et al., 1998; Miltenberger et al., 1999).

Another reason for the effectiveness of the video VSD-assisted BST could be the video VSD in the modeling stage of the BST. The findings reported by the studies conducted with video model (Gunby & Rapp, 2014; Ledbetter-Cho et al., 2021), only video VSD (Laubscher et al., 2019) and video model (Nikopoulos & Keenan, 2004; Olcay Gul et al., 2019) in the modeling stage of the BST were consistent with that premise. Furthermore, consistent with the social validity findings reported by the studies where videos were employed in instruction (Olcay Gul et al., 2019), the social validity findings of the present study demonstrated that individuals with ASD considered these videos entertaining and benefited more from video applications due to their interest in visual technological devices. It could be suggested that the availability of multiple examples, various scenarios, and high treatment reliability of video VSD-assisted BST were among the factors that led to effective outcomes within a short time. Due to the lack of studies conducted with the video VSD in the modeling stage of BST, the present study is unique in its contributions to BST, video VSD, and effective instruction literature.

The in-depth analysis of the effectiveness of video VSD-assisted BST in the instruction of skills to prevent online sexual abuse among adults with ASD revealed that these individuals could not display the target skill at the criteria level during the initial daily baseline sessions. It could be suggested that the instruction of the “no/non-response, “block” and “report” steps as a whole in the study could have prevented the achievement of the criteria by the participants in the initial sessions. Although this was more common for Dan, it was observed that Adam and Bruno rejected the message sent by the perpetrator in daily baseline sessions, and blocked the perpetrator; however, failed to report it to a trusted adult. Since it was reported in the literature that individuals with ASD experience difficulties in reporting security threats, this step is the most critical step (Girardi et al., 2021), a scoring system was developed in the present study to instruct all steps at once rather than instructing each step separately. The daily baseline session findings were consistent with the literature; however, these also suggested that the above-mentioned decision was appropriate for this sample.

One of the most important study findings that should be discussed was the follow-up and generalization findings. Providing natural opportunities to demonstrate acquired skills, selection of real-life abusive messages based on the views of their parents, and selection of unknown perpetrators of different age groups, genders, and physical traits helped the participants to maintain their acquired skills after the training and were effective in the generalization of acquired skills. It was expected that the selection of perpetrators in both genders would contribute to follow-up and generalization, as well as raise the awareness of the participants that online inappropriate messages could also come from individuals of the same gender. It should also be mentioned that the perpetrators were familiar individuals that the participants had met before but are unlikely to meet in the future or will never meet in the future in the generalization sessions. Thus, we aimed to determine how the participants would react to online inappropriate messages from familiar people and prevent future traumas and unethical situations by minimizing the possibility of encountering these perpetrators in daily life.

The social validity data collected with the interviews conducted with the participants, parents, and teachers of these individuals in the study demonstrated that all individuals expressed positive views on the instructed skills, the intervention, and the outcomes. The social validity data collected from the participants demonstrated that the participants not only exhibited the instructed skills but also learned these skills as rules. This finding demonstrated that the “no,” “block,” and “report” rules, which Miltenberger (2008) reported as common skills that could be exhibited against several security threats, were acquired by the participants. It could be suggested that this outcome could increase the exhibition of these skills by the participants in case of other threats and facilitate the acquisition of other safety skills in the future.

The teachers stated that the skills to prevent online sexual abuse instructed in the study were important for the safety of the participants. The statement by Bruno's teacher that to cope with sexual abuse, individuals should realize that the behavior of the abuser poses a security threat was quite significant. Studies in the literature reported that to cope with sexual abuse, it was imperative to realize that the abuser's behavior poses a security threat (Kenny & Wurtele, 2010). Social validity findings collected from the teachers supported this claim. However, it was surprising that Bruno's teacher also stated that the skills to prevent sexual abuse was not necessary for every student, and certain individuals with ASD could not learn these skills due to their performances. This finding was based on the least dangerous assumption that individuals with ASD could not learn certain skills. However, previous studies demonstrated that individuals with ASD could acquire the required skills when these are systematically instructed with adequate methods and techniques (Gevarter et al., 2020). Furthermore, studies indicated that individuals who attended safety skills training exhibited these skills when they encountered threats (Miltenberger, 2008). Also, studies reported that the acquisition of skills to prevent sexual abuse by individuals with ASD would improve participation in independent life and their quality of life; thus, the instruction of these skills is compulsory (Gevarter et al., 2020).

Two parents from whom social validity data were collected stated that they considered the instruction of skills to prevent online sexual abuse important for their children with ASD. Bruno's mother stated that individuals with ASD are more susceptible to sexual abuse due to the peculiarities of their disorder, and more information should be provided by educational institutions for the parents and students. Thus, after the study, the parents and teachers of the participants were informed about the recognition and prevention of sexual abuse, and the instruction of skills to prevent sexual abuse. The social validity data collected from the parents were consistent with the educational objectives of sexual education content, emphasized by institutions such as UNESCO (2018) and the National Guidelines Task Force (2004), which work on sexual education and aim to improve the social sexual education knowledge and skills, and emphasized the need for sexual education curricula at schools.

### Strengths and Limitations

It could be suggested that the study contributed to the literature in the related fields based on the issues considered in the planning process and the study findings. No previous studies are available on the effectiveness of BST in the instruction of skills to prevent sexual abuse among adults with ASD. Furthermore, there are no studies that employed the video VSD application in the modeling stage of BST and investigated the effectiveness of the intervention in the instruction of skills to prevent sexual abuse among adults with ASD. Thus, it could be suggested that both the dependent and independent variables of the study contributed to the literature. The review of the studies on the instruction of skills to prevent sexual abuse with BST revealed that these studies were conducted with 28–47 years old female participants with intellectual disabilities (Egemo-Helm et al., 2007; Lumley et al., 1998; Miltenberger et al., 1999), while no studies were conducted with male participants and individuals with ASD. Also, the present study was conducted with 21–23-year-old individuals with ASD, for whom the literature is quite limited. Thus, it could be suggested that the research contributed to the literature in terms of gender, diagnosis, and age properties. Finally, it could be suggested that the collection of social validity data from the participants, the teachers, and the parents of the participants was among the strengths of the study. In addition to the literary contributions of the study, it could be suggested that it would guide and inform the parents of individuals with ASD, specialists, and teachers who work with these individuals, on the acquisition of skills to prevent sexual abuse by these individuals.

Despite the above-mentioned originality and strengths of the study, it also has certain limitations. The instruction of “no/no response,” “block,” and “report” steps were instructed only on the WhatsApp application was the main limitation of the present study. Also, although abuse scenarios that the participants are likely to encounter in daily life were enacted in the study, since the intervention did not include any other type of online abuse, this could be considered as another limitation of the study.

### Recommendations

Based on the present study findings, the following recommendations could be suggested for future research and practice. Further studies could be conducted on the effectiveness of video VSD, a technological application adequate for advanced research, in the instruction of safety skills such as prevention of sexual abuse, and the effectiveness of interventions conducted with video VSD and BST or only BST in the instruction of safety skills. Further studies could investigate long-term follow-up (e.g., 3 months) and generalization of skills to prevent online sexual abuse by individuals with ASD after video VSD-assisted BST, and the effectiveness of the intervention implemented by parents and teachers in instruction of these skills. In practice, courses could be organized to raise the awareness of parents and experts on the topic, to improve their knowledge and skills on effective and proven methods for the instruction of these skills, and to raise the awareness of individuals with ASD about the threats to their personal safety and improve their performance in safety skills.
